# Questioning the Safety of Calcidiol in Hemodialysis Patients

**DOI:** 10.3390/nu11050959

**Published:** 2019-04-26

**Authors:** Ricardo Villa-Bellosta, Ignacio Mahillo-Fernández, Alberto Ortíz, Emilio González-Parra

**Affiliations:** 1 Renal Division, Fundación Instituto de Investigación Sanitaria Fundación Jiménez Díaz (FIIS-FJD), Avenida Reyes Católicos 2, 29040 Madrid, Spain; AOrtiz@fjd.es; 2 Biostatistics and Epidemiology Unit, Fundación Instituto de Investigación Sanitaria Fundación Jiménez Díaz (FIIS-FJD), 29040 Madrid, Spain; IMahillo@fjd.es; 3 Fundación Renal, Íñigo Álvarez de Toledo, 28003 Madrid, Spain; 4 Renal Division, IIS-Fundación Jiménez Díaz y Facultad de Medicina, Universidad Autónoma de Madrid, 28049 Madrid, Spain

**Keywords:** calcidiol, vitamin D, mortality, safety, dialysis

## Abstract

Background. Epidemiological studies have suggested a survival benefit for hemodialysis patients on paricalcitol or calcitriol, but nutritional vitamin D supplementation of patients already on vitamin D receptor (VDR) activators is controversial. Methods. This observational retrospective cohort study was conducted with prospectively collected data from all consecutive patients with chronic kidney disease (CKD) who underwent hemodialysis under routine clinical practice conditions for two years. Results. Of the 129 patients, 89 were treated with calcidiol, paricalcitol, and/or calcitriol. The patients with any vitamin D formulation had higher serum concentrations of 25-hydroxy vitamin D and fibroblast growth factor-23 and tended to have higher mortality rates (42% *vs.* 25%, *p* = 0.07). On subgroup analysis, any calcidiol treatment or calcidiol combined with paricalcitol associated with significantly higher mortality rates than no treatment (47% and 62.5%, *p* = 0.043 and 0.008, respectively). The association between calcidiol/paricalcitol treatment and elevated mortality remained significant after adjusting for age, sex, diabetes, C-reactive protein, and hemodialysis vintage. Any calcidiol and calcidiol/paricalcitol treatment exhibited a dose-response relationship with mortality (*p* for trend: 0.002 and 0.005, respectively). Conclusions. These data draw attention to the hitherto unexplored safety of calcidiol supplementation in patients on hemodialysis, especially in those already on vitamin D. Until clinical trials demonstrate the safety and efficacy of this approach, caution should be exercised when prescribing these patients ≥0.5 calcidiol mg/month.

## 1. Introduction

Patients with chronic kidney disease (CKD) and end-stage renal disease (ESRD) frequently exhibit vitamin D deficiency (defined as serum 25-hydroxy vitamin D levels of <15 ng/mL) or insufficiency (defined as serum 25-hydroxy vitamin D levels of <30 ng/mL) [1]. Several studies also show that low serum 25-hydroxy vitamin D levels in ESRD patients associate with higher all-cause mortality rates [2,3]. Accumulating epidemiological evidence also supports the hypothesis that vitamin D deficiency may contribute to the very high mortality risk of hemodialysis patients [4,5]. In particular, hemodialysis patients with severe vitamin D deficiency have an increased risk of cardiovascular mortality; however, they are not at increased risk of non-cardiovascular mortality [6]. 

Higher 25-hydroxy vitamin D levels in patients with CKD associate with significantly higher survival [7], and a meta-analysis based on observational studies showed that therapy with 1,25-dihydroxyvitamin D and its analogs associated with reduced mortality in CKD patients, especially in patients with secondary hyperparathyroidism [8]. However, it remains to be established whether vitamin D supplementation could improve the mortality rates in patients with CKD and cardiovascular disease because a meta-analysis of 51 randomized clinical trials on the ability of vitamin D to prevent various cardiovascular outcomes in the general population showed that this therapy had no overall benefit associated with supplementation [9]. Similarly, a 2017 systematic review and meta-analysis of the existing randomized controlled trials on the effect of vitamin D supplementation on mortality in CKD found that these trials did not clearly support the notion that this therapy reduced the mortality of patients with CKD [10].

It is important to clearly determine the role of vitamin D supplementation in CKD management because vitamin D overdosing can occur, especially in patients who are prescribed several vitamin D formulations or analogs simultaneously. As pediatricians are well aware, vitamin D overdosing can be deleterious [11]. Hypercalcemia is the hallmark of vitamin D intoxication. While it generally only occurs when the circulating 25-hydroxy vitamin D levels are consistently above 150 ng/mL [12], recent reports have led to concern that lower concentrations can lead to subclinical intoxication that is associated with adverse outcomes [12,13] such as dose-dependent effects on soft tissue calcification [14,15], hyperphosphatemia, hypercalcemia, increased matrix metalloproteinase levels, medial calcification, arterial stiffness, and left ventricular hypertrophy [16]. These risks of nutritional vitamin D overdosing may be particularly magnified in hemodialysis patients because they lack kidney regulatory mechanisms, obtain a positive calcium balance after each dialysis session, and are often simultaneously being treated with vitamin D receptor (VDR) activators. In this context, a recent small trial (30 patients) showed that low dose cholecalciferol (25 000 IU every 2 weeks) therapy for 1 year was safe in hemodialysis patients, some of whom were also receiving VDR activators. In particular, the therapy did not adversely affect the calcium, phosphorus, or parathyroid hormone (PTH) levels or promote vascular calcification [17]. However, the trial was too small and short to confirm the long-term safety of this approach. In addition, the trial was on cholecalciferol, whose pharmacology differed from that of calcidiol (25-hydroxy vitamin D), which is the nutritional vitamin D supplement that is recommended by the Spanish Society of Nephrology (SEN) CKD-MBD guidelines for increasing the serum 25-hydroxy vitamin D levels to >30 ng/mL [18].

The present observational retrospective cohort study was conducted to assess the association between different vitamin D therapies (calcidiol, calcitriol, and/or paricalcitol) with mortality in hemodialysis patients in a routine clinical practice setting. 

## 2. Materials and Methods

### 2.1. Patient Population

This observational retrospective cohort study was conducted according to the tenets of the Declaration of Helsinki and its revisions and was approved by the Ethics Committee of Research of University Hospital Fundación Jiménez Díaz (FJD-07-12). All participants signed the informed consent.

The study population consisted of all consecutive patients with CKD who underwent hemodialysis for two years in our academic tertiary care center (University Hospital Fundación Jiménez Díaz) in Madrid, Spain. Patients were included if they were stable adults (age >18 years) who were capable of informed consent and were on chronic hemodialysis, had a dialysis vintage of >6 months, and had a life expectancy of >6 months. Patients were excluded if they did not consent, had positive serology for hepatitis B surface antigen, hepatitis C virus, and/or human immunodeficiency virus, or had a life expectancy of <6 months. Patients transplanted during the study were also excluded.

All patients were treated according to routine clinical practice. All underwent conventional hemodialysis, thrice weekly for 4 hours, using a high-flux helixone dialyser (Fresenius; CUF, 59 mL/h/mmHg; surface, 1.8 m^2^). The acetate-acidified dialysate composition was 1.5 mmol/L calcium, 35 mmol/L bicarbonate, 0.75 mmol/L potassium, 0.5 mmol/L magnesium, and 140 mmol/L sodium. 

The variables that were retrieved retrospectively from the medical records of the patients included analytical and nutritional variables and mortality at the end of the two-year observational period. Overall mortality was the primary study outcome. All data were prospectively collected. 

### 2.2. Biochemical Variables

Biochemical variables were measured using an ADVIA CENTAUR 2400 autoanalyzer according to the manufacturer’s protocols. FGF-23 (C-terminal) was determined by ELISA (Immu-topics, USA) using two polyclonal antibodies that were specific for the C-terminus of FGF-23 (the intra-assay and inter-assay coefficients of variation were <1.7% and <3.5%, respectively; test sensitivity was 1.5 RU/mL). PTH was measured using a second generation electrochemiluminescence method with an Elecsys autoanalyzer (Roche). Total 25-hydroxy vitamin D (D2 and D3) levels were determined by electrochemiluminescence in an Elecsys autoanalyzer (Roche).

### 2.3. Statistical Analyses

The Kolmogorov–Smirnov test was used to assess the normality of the data. For variables with normally distributed data, the mean and SD was calculated and a Student’s t-test was used to compare patient groups. For variables with non-normally distributed data, the median and interquartile range was calculated and the Mann–Whitney U test was used to compare patient groups. To compare the vitamin D treatment groups in terms of mortality rates, the Chi-squared test or Fisher’s exact test was used. Mortality comparisons of patients who did not receive vitamin D with patients who received a combination of calcidiol and paricalcitol were adjusted by several potential confounders. These adjustments were made using logistic regression models.

In total, 129 patients who were undergoing hemodialysis in a single center were monitored for changes in analytical and nutritional variables and mortality during two years of follow-up.

## 3. Results

Table 1 shows the general characteristics of the study population at baseline (the start of the two-year observational period). The age and sex distribution of the cohort were consistent with the demographics of the general hemodialysis population in Spain [19]. Vitamin D therapy, defined as any vitamin D or Vitamin D receptor activator (calcidiol, calcitriol, or paricalcitol) formulation, was prescribed in 89 of the 129 (68.9%) patients at baseline. The patients on any vitamin D therapy did not differ significantly from the patients who were not on vitamin D therapy in terms of age, sex, or prevalence of diabetes (Table 1). The two groups also did not differ in terms of serum C-reactive protein (CRP), albumin, and hemoglobin (Hb) levels. However, the two groups did differ significantly in terms of several mineral and bone metabolism variables. Specifically, the patients on any vitamin D therapy had significantly higher 25-hydroxy vitamin D levels than the patients who were not on vitamin D (mean ± standard deviation [SD]: 30.26 ± 17.16 *vs.* 17.36 ± 9.77 ng/mL; *p* < 0.001). They also had significantly higher serum fibroblast growth factor (FGF)-23 levels (median [interquartile range]: 1207 (3367) *vs.* 603.5 (919.2) RU/mL; *p* < 0.043). The two groups did not differ in terms of serum PTH, phosphate, or calcium levels.

Table 2 shows the mortality of the patients who received the different vitamin D formulations (calcidiol, calcitriol, and/or paricalcitol) alone or in combination. Prescription of any vitamin D therapy tended to associate with higher mortality in the hemodialysis patients. Specifically, at the end of the two-year study period, 41.6% of the patients on any vitamin D therapy died whereas only 25% of the patients who were not on vitamin D therapy died (*p* = 0.07) (Table 2). Subgroup analysis then showed that the calcidiol/paricalcitol combination group had a significantly higher mortality rate (10/16, 62.5%) than the patients who were not on vitamin D (10/40, 25%; *p* < 0.008). A sensitivity analysis in which all patients who received a specific vitamin D formulation (either alone or in combination) were grouped showed that patients on calcidiol, either alone or in combination, had a significantly higher risk of death (27/57, 47.4%) than the patients who were not treated with vitamin D (10/40, 25%; *p* < 0.043). However, patients on any calcitriol combination and on any paricalcitol combination also had mortality rates that exceeded 40% (Table 2). 

**Figure 1:**
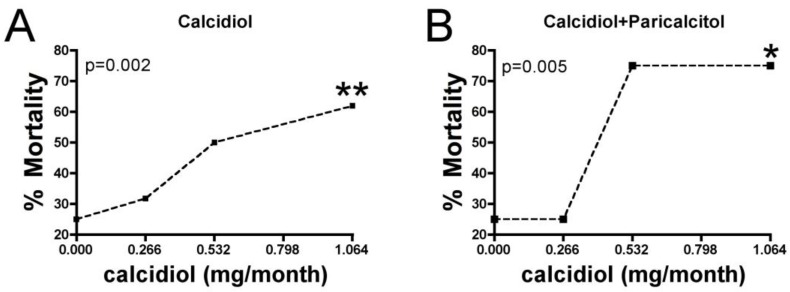
**Mortality according to calcidiol dose**. (**A**) Mortality in hemodialysis patients on any calcidiol treatment showed a significant dose-response association with calcidiol dose (p for trend 0.002) for doses of 0.266 (mortality 8/22), 0.532 (7/14) and 1.064 (12/21) mg/month. (**B**) Mortality in hemodialysis patients on calcidiol and paricalcitol also showed a significant dose-response association with calcidiol (*p* for trend 0.005) for doses of 0.266 (1/4), 0.532 (3/4) and 1.064 (6/8) mg/month. Mortality for controls (0 µg/month calcidiol) was 10/40. Chi-squared trend test was used to obtain the p value for linear trend, shown in the panel. Chi-squared test or Fisher’s exact test was used to compare the different doses with respect to the control. * *p* < 0.05; ** *p* < 0.001 vs no calcidiol.

In addition, the increased risk of death in patients on calcidiol (alone or in combination) or in patients on calcidiol in combination with paricalcitol exhibited a significant dose response (*p* for trend: 0.002 and 0.005, respectively; Figure 1). By contrast, of the patients on any vitamin D formulation, patients on paricalcitol alone had the lowest mortality rate (5/21, 23.8%).

Analysis of the serum levels of 25-hydroxy vitamin D in the different subgroups of patients on vitamin D showed that the patients on any vitamin D (including calcidiol, and combinations) had significantly higher levels of 25-hydroxy vitamin D (*p* < 0.001) than the patients who were not on vitamin D (Table 2 and Table 3). The patients on any paricalcitol also had significantly higher serum calcium levels than the patients who were not on vitamin D therapy. This was largely due to the patients on paricalcitol plus calcidiol, who had the highest serum calcium levels of all treated groups (Table 3). The patients receiving any paricalcitol also had significantly higher FGF-23 levels than the patients who were not on vitamin D. It should be noted that Chonchol M et al. found that high FGF-23 levels and low 25-hydroxy vitamin D levels were associated with higher mortality in hemodialysis patients [20]. However, it is unlikely that high FGF-23 and/or low 25-hydroxy vitamin D levels explain why patients on any vitamin D therapy, especially patients on calcidiol alone or in combination with paricalcitol, had higher mortality rates: Although patients with FGF-23 levels that were above the median (825 RU/mL) did tend to have a higher mortality rate (28/64, 43.75%) than patients with lower-than-median FGF-23 levels (18/63, 28.6%), this difference did not achieve statistical significance (*p* = 0.075). Moreover, the patients with 25-hydroxy vitamin D values above or below the median (23.3 ng/mL) did not differ in terms of mortality rates (data not shown). 

Subgroup analyses of the patient groups who received calcitriol, calcidiol, and/or paricalcitol also showed that those with above-median levels of FGF-23 did not have higher mortality rates than those with below-median levels of FGF-23 (Table 4).

The patients on a combination of paricalcitol and calcidiol had significantly higher hemodialysis vintage and C-reactive protein levels than the patients who did not receive vitamin D therapy (Table 3). However, after adjustment for these variables, the association between calcidiol/paricalcitol treatment and higher mortality remained significant (Table 5). In addition, the association between mortality and calcidiol/paricalcitol treatment remained significant after adjustment for age, sex, and presence of diabetes at baseline (Table 5).

## 4. Discussion

The main finding of this study was that, under routine clinical practice conditions, patients on hemodialysis who took calcidiol supplements, especially on top of VDR activators, had a higher risk of mortality than patients who did not receive vitamin D therapy. This safety signal, if confirmed, may require changes to clinical guidelines such as the current SEN CKD-MBD guidelines, which recommend that hemodialysis patients should receive calcidiol supplementation to maintain their 25-hydroxy vitamin D levels above 30 ng/mL [18].

The present study showed that any vitamin D supplementation in hemodialysis patients tended to be associated with higher mortality. However, the highest mortality was found in patients receiving a combination of calcidiol and paricalcitol. By contrast, patients on paricalcitol alone had the lowest mortality. Thus, calcidiol therapy may explain why any vitamin D supplementation associates with higher mortality. Indeed, we observed that “any calcidiol” (i.e., calcidiol alone or in combination with paricalcitol or calcitriol) exhibited a dose-response association with mortality. This dose response was also observed for patients specifically receiving the calcidiol/paricalcitol combination. This further highlights the possibility that calcidiol treatment contributes to the higher mortality of hemodialysis patients relative to the general population. It should be noted that randomized placebo-controlled trials that explore the effect of calcidiol supplementation on the mortality of hemodialysis patients were not been reported. Thus, from an evidence-based medicine point of view, the safety of calcidiol therapy in hemodialysis patients has not been established. 

It should be noted that the patients on calcitriol in the study also had a high mortality. However, the sample size of these patients was too low to reach any meaningful conclusions about the safety of this formulation in hemodialysis patients.

Notably, Wolf et al. [1] showed that, while low 25-hydroxy vitamin D levels in patients with CKD and ESRD were associated with increased mortality, there was a significant interaction between vitamin D levels, subsequent active vitamin D therapy, and survival. Specifically, compared with the patients with the lowest 25-hydroxy vitamin D levels who were untreated, the patients with low 25-hydroxy vitamin D who did receive active vitamin D therapy had a significantly lower risk of early mortality.

The mechanisms underlying the high mortality of the hemodialysis patients on calcidiol remain unclear. We initially speculated that FGF-23 levels may have been involved. This hormone is secreted by osteocytes and osteoblasts and regulates phosphate and vitamin D homeostasis. In CKD, FGF-23 concentrations increase progressively [21,22,23]. FGF-23 decreases plasma levels of calcitriol by downregulating its synthesis and enhancing its degradation [21,23]. High plasma FGF-23 levels are associated independently with endothelial dysfunction, arterial stiffness, left ventricular hypertrophy, progression of renal disease, and mortality and cardiovascular events [24]. In a seminal study, Collins et al. [25] showed that, in patients with hypoparathyroidism or pseudohypoparathyroidism, calcitriol therapy not only elevates serum vitamin D levels, but also increases serum FGF-23 levels. Thus, we asked whether calcidiol therapy elevated mortality by increasing serum FGF-23 levels. We found indeed that, compared with the patients who did not receive vitamin D therapy, patients receiving any vitamin D supplementation had significantly higher serum FGF-23 levels, although the two groups did not differ in serum phosphate, calcium, and PTH levels. However, subgroup analysis then showed that, compared with untreated patients, the patients on any calcidiol treatment did not differ from the patients who were not on vitamin D therapy in terms of FGF-23 or calcium levels, despite the fact that these patients had the highest mortality rates (47.4%). By contrast, paricalcitol treatment alone, which was associated with much lower mortality rates (23.8%), associated with higher serum FGF-23 and calcium levels. Thus, none of the CKD-MBD variables that we explored appeared to explain the association between calcidiol treatment in dialysis patients and increased mortality. It is possible that the increased mortality in the any-calcidiol group may instead relate to subclinical vitamin D toxicity; however, validated diagnostic tools for this toxicity have not yet been developed [12,13].

The study has some limitations that should be acknowledged. The sample size was relatively low, and the observational nature of the study precluded any conclusion regarding causality (*n* = 129). In this regard, while there may be a causal relationship between calcidiol treatment and increased mortality in hemodialysis patients, assessment of causality requires an interventional study and additional explanations are possible. One potential explanation is confusion by indication. In other words, patients who were at a higher risk of death may have been more likely to be prescribed calcidiol, possibly because they had lower serum 25-hydroxy vitamin D levels and such low levels are associated with a higher risk of death. However, this is unlikely because the intervention corrected the low serum 25-hydroxy vitamin D levels: The patients on calcidiol had higher 25-hydroxy vitamin D levels than the patients who were not on vitamin D. Several other potential explanations were excluded by the fact that the calcidiol and untreated patients did not differ in the prevalence of several key predictors of mortality, namely, male sex and diabetes; moreover, the association between calcidiol and mortality remained significant after adjustment for these and other variables. 

A key strength of this study is that, since it was performed in a single center, the standard of care was the same for all patients. Another strength was that the data were collected prospectively.

## 5. Conclusions

In conclusion, our data draw attention to the hitherto unexplored safety of calcidiol supplementation in patients on hemodialysis, especially in those who are already on some sort of vitamin D therapy. Studies in larger multicenter cohorts that test these findings are warranted. In the meantime, we urge caution when prescribing hemodialysis patients doses of calcidiol that are equal to or higher than 0.5 mg/month (30.000 IU/month). 

## Figures and Tables

**Table 1:** Demographic, biochemical and clinical characteristics of the study population.

Variable	All *n* = 129	No Vitamin D *n* = 40	Any Vitamin D *n* = 89	*p*
Age (years)	70.0 (57.0, 77.0)	68.5 (55, 75.2)	70 (57, 80)	0.307
Female, *n* (%)	59 (46%)	17 (43%)	42 (47%)	0.761
Diabetes, *n* (%)	28 (22%)	10 (25%)	18 (20%)	0.706
Dry weight (Kg)	65.6 ± 14.5	68.1 ± 14.6	64.5 ± 14.4	0.207
HD vintage (years)	3.00 (2.00, 5.00)	2.00 (1.75, 5.00)	3.50 (2.00, 6.00)	0.074
Weekly HD (hours)	12.0 (12.0, 12.0)	12.0 (12.0, 12.0)	12.0 (12.0, 12.0)	0.886
PINP (µg/L)	251 (161, 441)	255 (167, 382)	242 (162, 461)	0.931
CTX (pg/mL)	1.77 (1.27, 2.42)	1.89 (1.24, 2.43)	1.77 (1.28, 2.39)	0.981
PTH (pg/mL)	204 (118, 370)	204 (148, 366)	202 (114, 369)	0.670
25 OH Vit D (ng/ml)	26.3 ± 16.4	17.4 ± 9.8	30.3 ± 17.2	<0.001
FGF-23 (RU/ml)	825 (384, 3340)	603 (353, 1273)	1207 (464, 3831)	0.043
Ca (mg/dl)	9.21 ± 0.77	9.04 ± 0.84	9.28 ± 0.72	0.106
P (mg/dl)	4.48 ± 1.44	4.30 (3.80, 5.20)	4.40 (3.70, 5.50)	0.927
ALP (UI/l)	109 (89, 138)	106 (92.5, 128)	110 (89, 148)	0.421
Hb (g/dl)	11.9 ± 1.19	11.8 ± 1.82	11.9 ± 1.68	0.706
Urea (mg/dl)	119 ± 37.2	123 ± 39.2	117 ± 36.4	0.407
Total protein (g/dl)	6.56 ± 0.69	6.54 ± 0.82	6.57 ± 0.62	0.822
Albumin (g/dl)	3.64 ± 0.48	3.67 ± 0.58	3.62 ± 0.43	0.594
CO_2_ (mEq/L)	21.4 ± 3.57	20.6 ± 3.54	21.7 ± 3.56	0.135
CRP (mg/L)	0.60 (0.25, 2.15)	0.60 (0.25, 1.57)	0.60 (0.25, 2.30)	0.594
Ferritin (ng/ml)	406 ± 237	388 ± 241	415 ± 237	0.550

Kolmogorov–Smirnov test was used to assess the normality of the data. For variables with normally distributed data, the mean, standard deviation (SD) and Student’s t-test were used to describe and compare the data. For variables with non-normally distributed data, the median, interquartile range (p25, p75), and Mann–Whitney test were used.

**Table 2:** Distribution of vitamin D formulations and mortality.

Vitamin D Therapy	Deceased, *n*	Total, *n*	Mortality, %	*p* Value vs No Vitamin D
NO	10	40	25.0	
YES *	37	89	41.6	0.07
Calcidiol only	15	37	40.5	0.146
Calcitriol only	5	11	45.5	0.264
Paricalcitol only	5	21	23.8	0.918
Calcidiol + calcitriol	2	4	50.0	0.297
Calcidiol + paricalcitol	10	16	62.5	0.008
Any calcidiol	27	57	47.4	0.043
Any calcitriol	7	15	46.7	0.189
Any paricalcitol	15	37	40.5	0.226

Chi-squared test or Fisher’s exact test was used for statistical analysis. * Calcidiol, calcitriol, or paricalcitol.

**Table 3:** Biochemical parameters and vitamin D therapy. For variables with normally distributed data, the mean, standard deviation (SD) and Student’s t-test were used to describe and compare the data. For variables with non-normally distributed data, the median, interquartile range (p25, p75), and Mann–Whitney test were used.

Variable	No Vitamin D	Any Paricalcitol		Any Calcidiol		Paricalcitol + Calcidiol		Paricalcitol Only		Calcidiol Only	
Age (years)	68.5 (55.0, 75.2)	70.0 (59.0, 80.0)	0.426	70.0 (57.0, 76.0)	0.572	70.0 (65.8, 74.5)	0.580	70.0 (57.0, 80.0)	0.475	69.0 (57.0, 76.0)	0.791
HD Vintage (years)	2.00 (1.75,5.00)	3.00 (2.00, 7.25)	0.087	4.00 (2.00, 7.00)	0.019	5.50 (3.00, 12.0)	0.003	2.50 (1.75, 4.00)	0.981	4.00 (2.00, 6.00)	0.073
HD h/week	12.0 (12.0, 12.0)	12.0 (12.0, 12.2)	0.565	12.0 (12.0, 12.0)	0.545	12.0 (10.9, 12.4)	0.857	12.0 (12.0, 12.0)	0.466	12.0 (12.0, 12.0)	0.459
Dry weight (kg)	68.1 ± 14.6	64.6 ± 15.1	0.313	64.6 ± 14.7	0.260	65.0 ± 17.9	0.518	64.3 ± 13.4	0.330	64.5 ± 12.5	0.251
25 OH Vit D (ng/ml)	17.4 ± 9.77	28.6 ± 16.1	0.001	35.4 ± 17.3	<0.001	35.6 ± 17.5	0.001	23.3 ± 12.8	0.049	36.2 ± 17.2	<0.001
Hb (g/dl)	11.8 ± 1.82	12.0 ± 2.05	0.609	12.0 ± 1.68	0.446	12.5 ± 2.00	0.169	11.6 ± 2.03	0.713	11.9 ± 1.42	0.758
Urea (mg/dl)	123 ± 39.2	111 ± 31.9	0.146	117 ± 38.3	0.443	102 ± 31.6	0.063	118 ± 31.2	0.595	122 ± 40.0	0.940
Total proteins (g/dl)	6.54 ± 0.82	6.52 ± 0.55	0.881	6.54 ± 0.60	0.984	6.47 ± 0.52	0.749	6.55 ± 0.58	0.951	6.59 ± 0.62	0.769
Albumin (g/dl)	3.67 ± 0.58	3.61 ± 0.49	0.603	3.60 ± 0.43	0.479	3.47 ± 0.52	0.235	3.71 ± 0.45	0.787	3.67 ± 0.39	0.986
Ca (mg/dl)	9.04 ± 0.84	9.52 ± 0.84	0.014	9.28 ± 0.75	0.148	9.71 ± 1.00	0.014	9.38 ± 0.68	0.119	9.15 ± 0.54	0.496
CO_2_ (mEq/L)	20.6 ± 3.53	21.2 ± 3.84	0.499	22.0 ± 3.76	0.075	21.8 ± 4.28	0.299	20.8 ± 3.51	0.900	21.9 ± 3.62	0.127
Ferritin (ng/ml)	387 ± 241	379 ± 229	0.879	446 ± 244	0.243	412 ± 214	0.722	354 ± 241	0.611	444 ± 253	0.320
PINP (µg/L)	255 (167, 382)	263 (176, 464)	0.549	257 (158, 451)	0.777	284 (217, 759)	0.235	228 (174, 460)	0.927	257 (167, 451)	0.732
CTX (pg/mL)	1.89 (1.24, 2.43)	2.01 (1.31, 2.61)	0.513	1.85 (1.36, 2.39)	0.826	2.10 (1.48, 2.50)	0.389	1.76 (1.18, 2.61)	0.804	1.79 (1.42, 2.39)	0.844
PTH (pg/ml)	204 (148, 366)	281 (143, 468)	0.315	195 (104, 366)	0.647	346 (230, 497)	0.175	274 (143, 465)	0.724	154 (98.2, 318)	0.225
P (mg/dl)	4.30 (3.80, 5.20)	4.10 (3.80, 5.90)	0.783	4.30 (3.70, 5.30)	0.909	4.00 (3.75, 5.38)	0.586	4.50 (3.80, 6.20)	0.970	4.30 (3.70, 5.30)	0.919
ALP (UI/l)	106 (92.5, 128)	116 (89.0, 163)	0.183	111 (87.0, 138)	0.684	122 (102, 165)	0.188	108 (89.0, 163)	0.383	108 (88.0, 136)	0.862
CRP (mg/L)	0.60 (0.25, 1.57)	0.25 (0.25, 2.20)	0.791	0.95 (0.25, 2.83)	0.170	1.55 (0.85, 2.57)	0.019	0.25 (0.25, 0.25)	0.093	0.60 (0.25, 2.65)	0.728
FGF23 (RU/ml)	603 (353, 1273)	1618 (788, 4768)	0.003	868 (392, 3422)	0.206	1939 (903, 6098)	0.023	1472 (788, 4768)	0.010	644 (372, 3047)	0.519

**Table 4:** Mortality in patients with 25-hydroxy vitamin D (VitD) and FGF-23 levels above or below the median (23.3 ng/mL and 825 RU/mL, respectively).

Group	Deceased/Total, *n* (%)	*p* Value
VitD low and FGF-23 low	8/31 (25.8%)	
VitD low and FGF-23 high	14/33 (42.4%)	0.256
VitD high and FGF-23 low	10/32 (31.2%)	0.842
VitD high and FGF-23 high	14/31 (42.2%)	0.185

**Table 5:** Calcidiol and paricalcitol combination and mortality.

Adjustment Variable	HR	(95% CI)	*p* Value
Age	4.01	(1.64–9.81)	0.002
Sex	3.27	(1.33–8.03)	0.010
DM	5.61	(2.00–15.7)	0.001
Age + Sex + DM	5.27	(1.79–15.6)	0.003
HD vintage (years)	3.77	(1.49–9.52)	0.005
25 OH Vitamin D levels (ng/mL)	4.87	(1.65–14.4)	0.004
Ca (mg/dL)	3.74	(1.45–9.61)	0.006
CRP (mg/L)	3.24	(1.29–8.13)	0.013
FGF-23 (RU/mL)	5.25	(2.11–13.1)	<0.001

Hazard Ratio (HR) and *p* value for mortality in patients on a combination of calcidiol and paricalcitol compared to patients who did not receive vitamin D treatment, after adjustment for the indicated variables using multivariable logistic regression models.
